# Depigmented-polymerised allergoids favour regulatory over effector T cells: enhancement by 1α, 25-dihydroxyvitamin D3

**DOI:** 10.1186/1471-2172-15-21

**Published:** 2014-05-29

**Authors:** Zoe L Urry, David F Richards, Cheryl Black, Maria Morales, Jerónimo Carnés, Catherine M Hawrylowicz, Douglas S Robinson

**Affiliations:** 1 Department of Allergy and Asthma, MRC and Asthma UK Centre for Mechanisms of Allergic Asthma, Guy’s Campus, King’s College London, London, UK; 2 Department of Research and Development, Laboratorios Leti, Tres Cantos, Madrid, Spain; 3 Leukocyte Biology Section, MRC and Asthma UK Centre for Mechanisms of Allergic Asthma, NHLI, Imperial College London, London, UK

**Keywords:** Allergen extract, Depigmented-polymerised, Immunotherapy, Regulatory T cell, Vitamin D

## Abstract

**Background:**

Allergen immunotherapy (SIT) is the only treatment for allergic disease capable of modifying disease long term. To reduce the risk of anaphylaxis from SIT, allergen-extracts have been modified by polymerisation with glutaraldehyde to reduce IgE binding. It is suggested that these allergoid extracts also have reduced T cell activity, which could compromise clinical efficacy. Effective SIT is thought to act through regulatory T cells (Tregs) rather than activation of effector T cells. There is no published data on the activity of modified extracts on Tregs.

**Results:**

We compared the capacity of modified (depigmented-polymerised) versus unmodified (native) allergen extracts of grass pollen and house dust mite to stimulate proliferation/cytokine production and to modulate Treg/effector T cell frequency in cultures of peripheral blood mononuclear cells (PBMC), from volunteers sensitised to both allergens in vitro. Depigmented-polymerised allergen extracts stimulated less proliferation of PBMC, and reduced effector cell numbers after 7 days in culture than did native extracts. However, the frequency of Foxp3+ Tregs in cultures were similar to those seen with native extract so that ratios of regulatory to effector T cells were significantly increased in cultures stimulated with depigmented-polymerised extracts. Addition of 1α, 25-dihydroxyvitamin D3 further favoured Treg, and reduced effector cytokine production, but not interleukin-10.

**Conclusions:**

Depigmented-polymerised allergen extracts appear to favour Treg expansion over activation of effector T cells and this may relate to their demonstrated efficacy and safety in SIT. 1α, 25-dihydroxyvitamin D3 further reduces effector T cell activation by allergen extracts and may be a useful adjuvant for SIT.

## Background

Specific allergen immunotherapy (SIT) is recommended for persistent allergic rhinoconjunctivitis and asthma resistant to pharmacotherapy, and is the only disease-modifying treatment in that benefit persists after stopping treatment [1,2]. However, subcutaneous injection of immunotherapy (SCIT) extracts carries a risk of anaphylaxis, whilst sublingual therapy (SLIT) requires daily treatment over three years. One approach to reducing the risk of anaphylaxis with SCIT is to modify allergen extracts to reduce IgE binding [3]. Such allergoids include extracts treated with acid (“depigmented”), prior to polymerisation with glutaraldehyde [4]. These extracts showed efficacy in clinical trials for both birch and grass pollen-related allergic rhinitis, and house dust mite-related asthma [5-8]. In these studies and large observational series, side effects were minimal [5-9]. The resulting depigmented-polymerised (Depig-pol) allergen extracts are of very large molecular mass (0.5-3 MDa as opposed to less than 100 KDa for native allergen extracts), and have at least 50 times reduced IgE binding in vitro in competition assays and in vivo in skin prick testing [4,10,11]. Analysis of sequences obtained by enzymatic digestion of Depig-pol and native extracts suggests conservation of major allergen sequences for both dust mite and pollen extracts [10,11], and in vivo immunogenicity studies show induction of IgG directed against the majority of major allergens upon treatment of patients with these extracts and in studies involving immunization of experimental animals [5-7,11].

Whilst allergoids have reduced IgE binding (allergenicity) which may improve safety, it has also been suggested that they have reduced T cell stimulating activity (immunogenicity) which could compromise efficacy [12]. Studies of T cell activity of allergoids in terms of proliferation of primary T cells, lines or clones, have suggested variable degrees of loss of ability to stimulate T cells, either generally or restricted to some epitopes [12-16]. However, the immunological efficacy of SIT most likely relates to induction of tolerance to allergens through interactions with regulatory T cells (Treg) [17]. Studies with unmodified allergen extracts suggest increased numbers and activity of both CD4^+^CD25^hi^Foxp3^hi^ T cells and IL-10 producing T cells after SIT treatment, together with induction of the IL-10-dependent antibody IgG4 [17]. Thus expansion of Tregs may be a more relevant activity to assess for allergen extracts for SIT.

In order to improve efficacy, reduce dosing and improve safety of allergen immunotherapy a number of adjuvants have been examined. In particular, there is much current interest in 1α, 25-dihydroxyvitamin D3, which can expand both induced IL-10-producing and Foxp3+ Treg in vitro, and improved effects of immunotherapy in animal models [18-21]. Vitamin D status and/or treatment of human volunteers with vitamin D derivatives correlated with or increased Foxp3+ T cell frequency in the periphery [19] and the airway in asthmatics [20] and IL-10 producing Treg in the periphery [18].

Here we have examined the relative stimulation of effector and regulatory T cells in vitro by unmodified and depigmented-polymerised allergen extracts, together with the effects of 1α, 25-dihydroxyvitamin D3.

## Results

### Depigmented-polymerised allergen extracts stimulate less T cell proliferation than native extracts, but similar cytokine production

**Figure 1:**
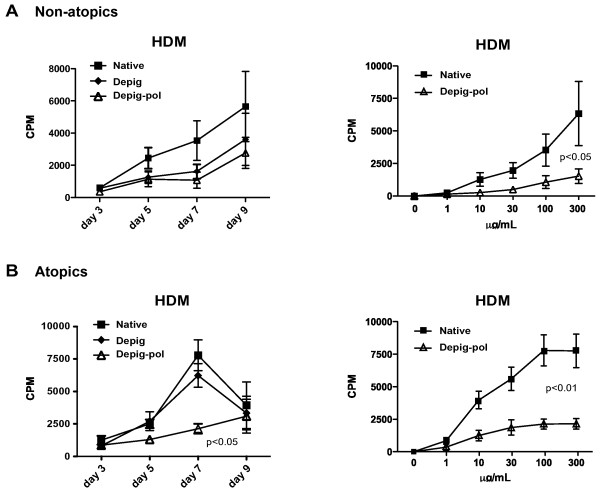
**Depigmented-polymerised house dust mite extract stimulates significantly less PBMC proliferation than unmodified native extract.** PBMC (2x10^6^/mL) were cultured with depigmented-polymerised (Depig-pol), depigmented (Depig) or native HDM extracts at different concentrations for different times: proliferation was assessed by thymidine incorporation, as described in Methods. N = 6. Statistical analysis by ANOVA. **A** Time course for proliferation of Depig-pol, Depig and native HDM extract-stimulated PBMC at a concentration of 100 μg/mL of each extract. p value for Depig-pol vs. native extract. **B** Dose-response for proliferation of Depig-pol, and native HDM extract-stimulated PBMC at day 7.

Production of depigmented-polymerised extracts involves two steps which might alter T cell reactivity (depigmentation: acid hydrolysis then dialysis to remove low molecular weight non-allergen material, then polymerisation with glutaraldehyde). Therefore initial timecourse and dose-response experiments were performed to assess proliferation of PBMC from atopic donors (Table 1) in cultures stimulated with native, depigmented and depigmented-polymerised (Depig-pol) house dust mite (HDM) and grass pollen (GRASS) extracts. Maximal proliferation was seen at day 7 for all extracts except for Depig-pol HDM extract which was highest at day 9 (Figure 1A). Note that PBMC viability was comparable between HDM native and Depig-pol stimulated cultures, 76.8% (+/- 23%) and 78.2% (+/- 26%), respectively. Data for some of the experiments with grass pollen extracts has been presented elsewhere [7].

**Table 1:** Volunteer characteristics

***Dermatophagoides pteronyssinus (HDM) Phleum pratense (Grass)***						
**Donor ID**	**IgE (kU/L)**	**CAPclass**	**SPT**	**IgE (kU/L)**	**CAPclass**	**SPT**
A03	3.85	3	4×4	9.83	3	10×6
A40	1.25	2	7×6	0.82	2	9×8
AR48	26	4	5×4	15.2	3	8×6
AR21	8.66	3	12×7	0.99	2	20×10
AR22	2.07	2	5×5	0.70	1	4×4
A58	6.75	3	6×5	1.31	2	5×4

Donor clinical characteristics are shown in brackets (AR = allergic rhinitis, A = asthma), specific IgE concentration and CAP class for each allergen, and size of skin prick test weal in mm.

Note equal proportions of male and female subjects were studied. Mean (range) of allergic patients was 38 years old (25 - 63 years); mean age of nonatopic individuals was 40 years old (30 - 47 years).

Proliferation for depigmented HDM and GRASS extracts was very similar to that seen for native extracts, but proliferation was signficantly less for Depig-pol extracts of both HDM and GRASS when compared to native unmodified extracts (Figure 1B and ref [7]). For this reason, further comparisons were between native and Depig-pol extracts.

**Figure 2:**
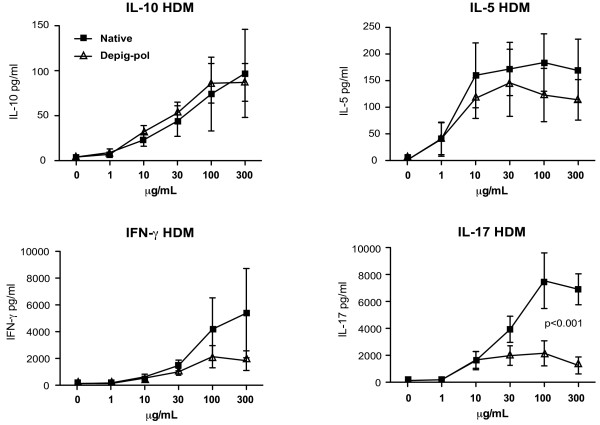
**Depigmented-polymerised house dust mite extract stimulates significantly reduced IL-17 production than unmodified native extract.** PBMC (2×10^6^/mL) were cultured with depigmented-polymerised (Depig-pol), depigmented (Depig) or native HDM extracts at different concentrations for different times: cytokine production was assessed by CBA, as described in Methods. N = 6. Statistical analysis by ANOVA. Dose-response for IL-10, IL-5, IFNγ and IL-17 production by Depig-pol, and native HDM extract-stimulated PBMC at day 5 (IL-10) or day 7 (IL-5, IFNγ and IL-17).

Cytokine production was also maximal at day seven for all cytokines except IL-10 which peaked at day 5 (data not shown). Seven days of culture was therefore used for further comparisons (except for IL-10). Production of IL-5, IL-10 and IL-13 did not differ significantly between cultures stimulated with native or depigmented-polymerised extracts of either HDM or GRASS (Figure 2, ref [7] and data not shown), whilst there was a non-significant trend for reduced IFNγ and significantly reduced IL-17 in depig-pol-stimulated cultures compared to native extract.

### Depigmented-polymerised allergen extracts expand regulatory T cells more than effector T cells in culture

**Figure 3:**
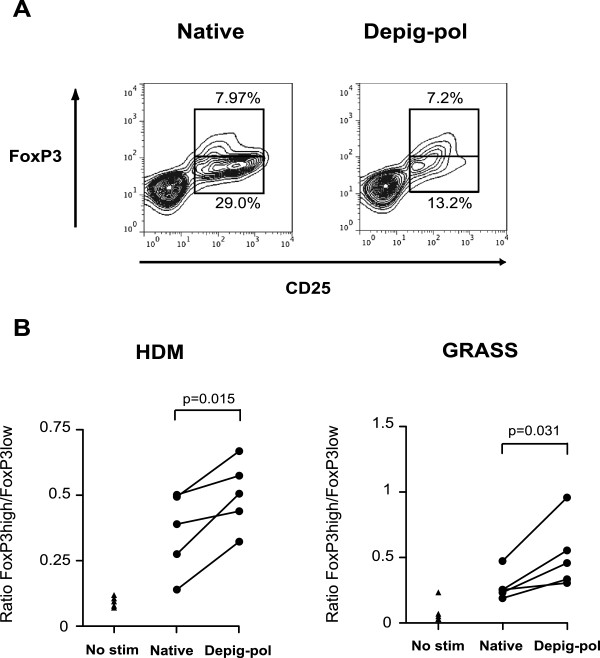
**Depigmented-polymerised allergen extracts favour regulatory T cell-phenotype over effector phenotype. A** Contour plots showing CD4^+^CD25^+^Foxp3^hi^ populations (regulatory phenotype) and CD4^+^CD25^+^Foxp3^low^ cells (effector phenotype) after 7-day culture with depigmented-polymerised (Depig-pol) or native HDM extracts. **B** Ratios of CD4^+^CD25^+^Foxp3^hi^ to CD4^+^CD25^+^Foxp3^low^ T cells from 7-day cultures stimulated with depigmented-polymerised or native extracts. N = 5, p values for paired *t*-test.

To examine effects of native and depigmented-polymerised extracts in stimulation of regulatory or effector cells we examined expression of CD25, Foxp3 and CD127 after 7 days of culture of PBMC. Gating strategy was as described, to identify cells that we have previously shown to be regulatory in cultures [19]. For both HDM and GRASS, Depig-pol extracts expanded similar numbers of CD4^+^CD25^+^Foxp3^hi^CD127^lo^ (regulatory T cells) compared to native extracts, but reduced numbers of CD4^+^CD25^+^Foxp3^lo^CD127^+^ (effector cells, Figure 3A). When expressed as a ratio of CD25^+^Foxp3^hi^ CD127^lo^ to CD25^+^Foxp3^lo^CD127^+^ cells this was significantly higher for Depig-pol extracts of both HDM and GRASS when compared to cultures stimulated with native extracts (Figure 3B).

### Addition of 1α, 25-dihydroxyvitamin D3 to cultures further favours CD25^hi^Foxp3^hi^CD127^lo^ T cells

**Figure 4:**
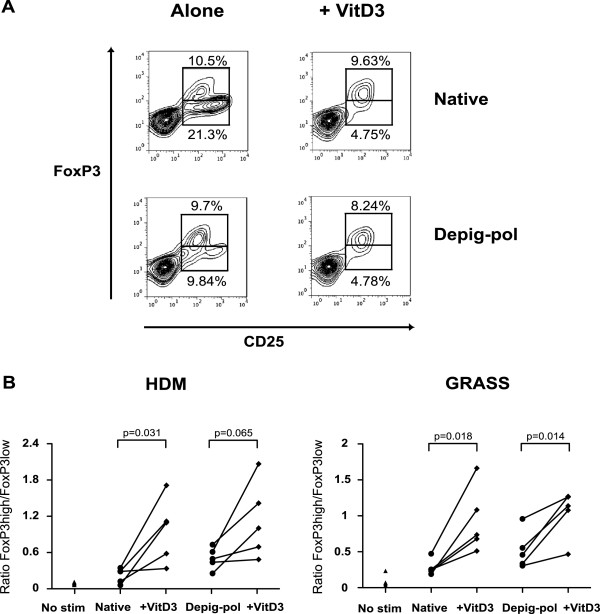
**1α, 25-dihydroxyvitamin D3 increases the ratio of regulatory to effector T cell phenotype. A** Contour plots showing CD4^+^CD25^+^FoxP3^hi^ populations and CD4^+^CD25^+^Foxp3^low^ T cells after 7 days culture with depigmented-polymerised (Depig-pol) or native grass pollen extracts with and without 1α, 25-dihydroxyvitamin D3 (10^-7^ M). **B** Ratios of Foxp3^hi^ to Foxp3^low^ T cells in cultures stimulated for 7 days with HDM or grass pollen with and without 1α, 25-dihydroxyvitamin D3. N = 5, analysis by paired *t*-test.

**Figure 5:**
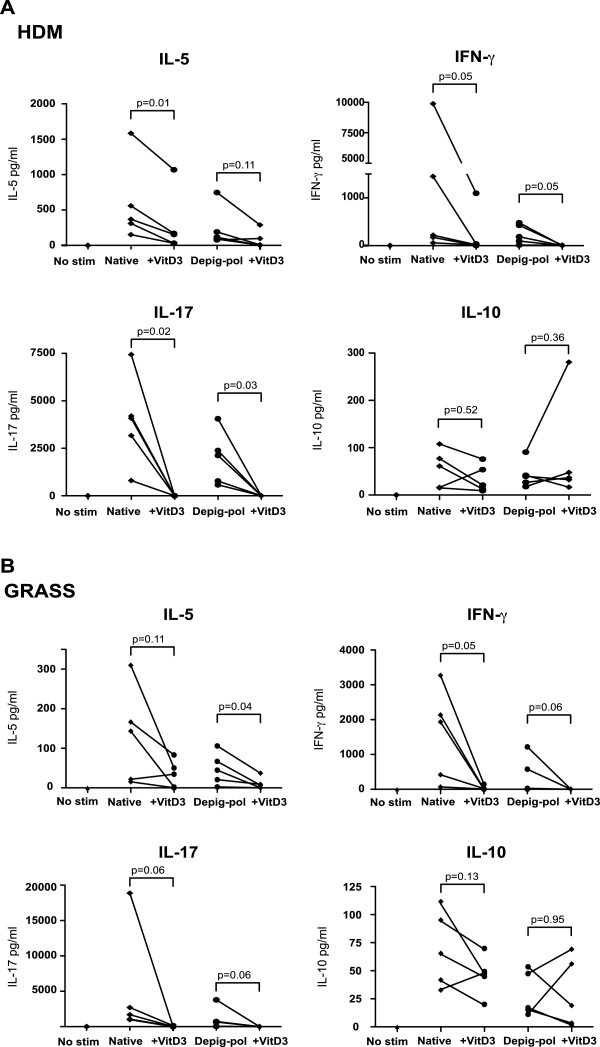
**1α, 25-dihydroxyvitamin D3 decreases production of effector cytokines but does not alter IL-10 production.** Cytokine production (by CBA) in 7-day PBMC cultures stimulated with **(A)** HDM or **(B)** Grass depigmented-polymerized (Depig-pol) or native extracts with and without 1α, 25-dihydroxyvitamin D3 (10^-7^ M). N = 5, analysis by paired *t*-test or Wilcoxon test.

Addition of 1α, 25-dihydroxyvitamin D3 at 10^-7^ M further reduced expansion of effector cells in cultures stimulated with both native and Depig-pol extracts of HDM and GRASS (Figure 4A), whilst numbers of CD4^+^CD25^+^Foxp3^hi^CD127^lo^ regulatory cells were not reduced. Thus the ratio of regulatory to effector cells after cultures was further increased by addition of 1α, 25-dihydroxyvitamin D3 (Figure 4B). In addition 1α, 25-dihydroxyvitamin D3 reduced effector cytokines, particularly IL-17 and IFNγ, but did not significantly affect IL-10 production (Figure 5 and data not shown).

## Discussion

These data suggest reduced proliferation and cytokine production (particularly IL-17 and IFNγ) by PBMC from atopic donors stimulated with Depig-pol extracts compared to native, unmodified allergen extracts for grass pollen and house dust mite. However, Depig-pol allergen extracts for grass pollen and HDM favoured expansion of regulatory T cells over effector T cells in vitro, and this balance was further tilted towards regulation by 1α, 25-dihydroxyvitamin D3. Thus Depig-pol allergen extracts may favour regulatory T cells over effector T cell activation, which might be expected to augment tolerance induction in SIT.

Several reports have previously examined T cell responses to allergoids [12-16]. All showed reduced proliferation of PBMCs stimulated with allergoid when compared to native allergen, and variable reductions in cytokine production. This difference in T cell proliferation between allergoid and native extract was suggested to vary with antigen presenting cell type, although this was not confirmed in a subsequent study [15,16]. Analysis of responses of T cell lines and clones suggested variable loss of T cell epitopes in allergoid extracts compared to native extracts [13-15]. Our data also suggests reduced proliferation and effector cytokine production by PBMC stimulated by Depig-pol extracts compared with native extract, and these findings were confirmed by reduced expansion of cells with effector phenotype in Depig-pol stimulated cultures. We would argue that reduced activation and expansion of effector T cells by Depig-pol extracts will actually be beneficial in the context of SIT, where effector T cell activation may contribute to side effects. This is demonstrated most graphically for peptide therapy where small allergen peptides which did not cross link IgE nonetheless activated effector T cells and lead to isolated late asthmatic reactions in patients [22]. We did not examine loss of T cell epitopes comparing Depig-pol extracts with native extracts, although previous mass spectroscopic analysis has suggested preservation of most major allergen sequences [10]. Even if some T cell epitopes were lost during the depigmentation and polymerisation process, we would argue that the critical activity for tolerance induction is expansion of regulatory T cells that work in both antigen-specific and non-antigen specific fashion, as shown by linked suppression to cat major allergen Fel d 1 peptides not included in the experimental peptide immunotherapy treatment in an animal model [23].

Although Depig-pol extracts stimulated reduced effector T cell expansion and activation compared to native extracts, numbers of regulatory T cells were similar, so the ratio of regulatory to effector T cells in cultures stimulated with Depig-pol extracts was significantly higher than for native extract. Activation requirements for effector versus regulatory T cells are incompletely understood, but we would suggest that antigen-presenting cell processing of and activation by these molecules of vastly different mass may be relevant in the case of Depig-pol versus native extracts [10,11].

Interestingly, our data both here and in previous studies [20] suggest that depigmented-polymerised extracts and 1α, 25-dihydroxyvitamin D3 both favour Foxp3^hi^ T cells (by reducing expansion of effector T cells). Initial reports suggested that 1α, 25-dihydroxyvitamin D3 acted to expand IL-10 producing T cells [18]. However, our recent data shows that 1α, 25-dihydroxyvitamin D3 can also expand Foxp3^hi^ Tregs [20]. The type of Treg emerging depended on the concentration of 1α, 25-dihydroxyvitamin D3 in cultures, and the cytokine milieu and essentially no co-expression of Foxp3 and IL-10 was observed, although both populations exhibited comparable suppressive activity [19,20]. The present findings are in keeping with that data in that a relatively higher concentration favoured Foxp3^hi^ Tregs (albeit 10^-7^ M here rather than 10^-6^ M in an anti-CD3 driven culture system), and in these conditions IL-10 producing T cells were not detected (data not shown). Our previous report suggested that the major effect of 1α, 25-dihydroxyvitamin D3 was to suppress proliferation of effector T cells and maintain Foxp3 expression by regulatory T cells: our current data agrees with this, as we show reduced effector T cells and cytokines, and a preserved Foxp3^hi^ Treg population [20].

SIT with depigmented-polymerised extracts, which have at least 95% reduced IgE binding compared to native allergen extract, has been shown to be clinically effective in reducing symptom scores for rhinoconjunctivitis and asthma with minimal side effects and rapid up-dosing [5-8]. Further studies are required to examine the effects of Depig-pol extracts on Tregs in vivo, and to determine whether 1α, 25-dihydroxyvitamin D3 can enhance tolerance in a clinical setting of SIT.

## Conclusion

Depigmented-polymerised allergen extracts appear to favour Treg expansion over activation of effector T cells to a greater extent than unmodified allergen extracts. 1α, 25-dihydroxyvitamin D3 further reduces effector T cell activation by both allergen extracts, and may be a useful adjuvant for SIT.

## Methods

### Volunteers and peripheral blood mononuclear cell (PBMC) culture

Atopic allergic volunteers were sensitized to both grass pollen and house dust mite (by skin prick tests and/or specific IgE). Clinical details are shown in Table 1. All had persistent AR and in 3 cases asthma. None was treated with systemic corticosteroids and all had stable disease controlled with topical treatment only. None of the volunteers had received immunotherapy. The study was approved by Guy’s Hospital Ethics Committee and all volunteers gave written consent. Skin prick tests were performed using Soluprick (ALK Horsholm, Denmark) and specific IgE was measured using immunoCAP (Phadia, Uppsala, Sweden). PBMC were separated by Lymphoprep density centrifugation (Axis-Shield, Oslo, Norway), then cultured in RPMI 1640 media with L-glutamine, gentamicin (all from Invitrogen, Paisley, UK), and 5% AB human serum (PAA, Pasching, Austria) in triplicate in 96 well plates at 2×10^6^ cells/ml for 7 days with a range of concentrations of allergen extracts or medium alone in a 5%CO_2_ incubator at 37°C. Proliferation was assessed by incorporation of titrated thymidine (1 μCi; added 16 hours before analysis) and cytokine production by cytometric bead array (BD Biosciences, Oxford UK). Depigmented-polymerised *Phleum pratense* (Timothy grass) pollen and *Dermatophagoides pteronyssinus* (house dust mite, HDM) extracts were compared with native, unmodified allergen extracts, and initially with depigmented non-polymerised extracts (all prepared as described by Laboratorios Leti, Tres Cantos, Spain, 4).

Assessment of regulatory and effector cells in culture and effect of 1α, 25 hydroxyvitamin D3 Flow cytometry was used to examine the relative numbers of T cells with regulatory or effector phenotype after 7 days culture as previously described [19] (CD4^+^CD25^hi^CD127^lo^Foxp3^hi^ for regulatory T cells, and CD4^+^CD127^+^CD25^+^Foxp3^lo^ for effector T cells). All antibodies were from BD Biosciences (Oxford, UK) except that for Foxp3, which was from EBiosciences (Hatfield, UK). We have previously used this gating strategy to identify regulatory T cells after culture, and confirmed suppressive activity of these cells by re-culture of sorted cells [19]. We added 1α, 25-dihydroxyvitamin D3 (BIOMOL Research Labs, Plymouth Meeting PA, USA) to further cultures of PBMC from atopic donors with both depigmented-polymerised and native allergen extracts. Pilot experiments showed the optimal dose of 1α, 25-dihydroxyvitamin D3 for expansion of Foxp3^+^ T cells to be 10^-7^ M in this culture system (data not shown).

### Statistical analysis

Data for proliferation and cytokine production was compared by ANOVA for dose response data for native and Depig-pol extracts. Values for regulatory/effector cell ratios and cytokines were compared by paired or unpaired *t*-test or Mann Whitney U/Wilcoxon tests as appropriate after testing for normality of distribution. All statistics were performed using GraphPad Prism (La Jolla, CA, USA).

## Competing interests

Authors declare no competing financial interests.

## Authors’ contributions

CMH and DS conceived and secured funding for the project. ZLU, DFR, CB, MEM, JC, CMH and DSR contributed to the design of, performed and analyzed data from all experiments. ZLU, DSR and CMH designed the study. ZLU, DR and CB performed all experiments. MEM measured serum allergen specific IgE and with JC supervised preparation of extracts. ZLU, DFR, DSR and CMH analysed data and ZLU, DSR and CMH wrote the manuscript. All authors read and approved the final manuscript.

## References

[B1] Bousquet J Lockey R Maling HJ Allergen immunotherapy: therapeutic vaccines for allergic diseases. A WHO Position paper J Allergy Clin Immunol 1998 102 558 562 10.1016/S0091-6749(98)70271-4 9802362

[B2] Alvarez-Cuesta E Bousquet J Canonica GW Durham SR Malling HJ Valovirta E Standards for practical allergen-specific immunotherapy Allergy 2006 61 Suppl 82 1 20 16930249 10.1111/j.1398-9995.2006.01219_1.x

[B3] Grammer LC Shaughnessy MA Patterson R Modified forms of allergen immunotherapy J Allergy Clin Immunol 1985 76 397 401 10.1016/0091-6749(85)90661-X 3926854

[B4] Casanovas M Gómez MJ Carnés J Fernández-Caldas E Skin tests with native, depigmented and glutaraldehyde polymerized allergen extracts J Investig Allergol Clin Immunol 2005 15 30 36 15864880

[B5] Pfaar O Robinson DS Sager A Emuzyte R Immunotherapy with depigmented-polymerized mixed tree pollen extract: a clinical trial and responder analysis Allergy 2010 65 1614 1621 10.1111/j.1398-9995.2010.02413.x 20645937

[B6] Höiby AS Strand V Robinson DS Sager A Rak S Efficacy, safety, and immunological effects of a 2-year immunotherapy with Depigoid birch pollen extract: a randomized, double-blind, placebo-controlled study Clin Exp Allergy 2010 40 1062 1070 10.1111/j.1365-2222.2010.03521.x 20642579

[B7] Pfaar O Urry Z Robinson DS Sager A Richards D Hawrylowicz CM Bräutigam M Klimek L A randomized placebo-controlled trial of rush preseasonal depigmented polymerized grass pollen immunotherapy Allergy 2012 67 2 272 279 10.1111/j.1398-9995.2011.02736.x 22107266

[B8] Garcia-Robaina JC Sanchez I de la Torre F Fernandez-Caldas E Casanovas M Successful management of mite-allergic asthma with modified extracts of *Dermatophagoides pteronyssinus* and *Dermatophagoides farinae* in a double-blind, placebo-controlled study J Allergy Clin Immunol 2006 118 1026 1032 10.1016/j.jaci.2006.07.043 17088125

[B9] Pfaar O Klimek L Sager A Bräutigam M Safety of a depigmented, polymerized vaccine for the treatment of allergic rhinoconjunctivitis and allergic asthma Am J Rhinol Allergy 2010 24 220 225 10.2500/ajra.2010.24.3437 20167138

[B10] Carnés J Himly M Gallego M Iraola V Robinson DS Fernández-Caldas E Briza P Detection of allergen composition and in vivo immunogenicity of depigmented allergoids of Betula alba Clin Exp Allergy 2009 39 426 434 10.1111/j.1365-2222.2008.03132.x 19134021

[B11] Gallego MT Iraola V Himly M Robinson DS Badiola C García-Robaina JC Briza P Carnés J Depigmented and polymerised house dust mite allergoid: allergen content, induction of IgG4 and clinical response Int Arch Allergy Immunol 2010 153 61 69 10.1159/000301580 20357486

[B12] Lund L Henmar H Würtzen PA Lund G Hjortskov N Larsen JN Comparison of allergenicity and immunogenicity of an intact allergen vaccine and commercially available allergoid products for birch pollen immunotherapy Clin Exp Allergy 2007 37 564 71.32 10.1111/j.1365-2222.2007.02687.x 17430354

[B13] Dormann D Ebner C Jarman ER Montermann E Kraft D Reske-Kunz AB Responses of human birch pollen allergen-reactive T cells to chemically modified allergens (allergoids) Clin Exp Allergy 1998 28 1374 1383 10.1046/j.1365-2222.1998.00407.x 9824410

[B14] Kahlert H Stuwe H-T Cromwell O Fiebig H Reactivity of T cells with grass pollen allergen extract and allergoid Int Arch Allergy Immunol 1999 120 146 157 10.1159/000024233 10545769

[B15] Kahlert H Grage-Griebenow E Stüwe HT Cromwell O Fiebig H T cell reactivity with allergoids: influence of the type of APC J Immunol 2000 165 1807 1815 10.4049/jimmunol.165.4.1807 10925258

[B16] Kalinski P Lebre MC Kramer D De Jong EC Van Schijndel JW Kapsenberg ML Analysis of the CD4+ T cell responses to house dust mite allergoid Allergy 2003 58 648 656 10.1034/j.1398-9995.2003.00240.x 12823126

[B17] Akdis CA Akdis M Mechanisms of allergen-specific immunotherapy J Allergy Clin Immunol 2011 127 18 27 10.1016/j.jaci.2010.11.030 21211639

[B18] Urry Z Xystrakis E Richards DF McDonald J Sattar Z Cousins DJ Corrigan CJ Hickman E Brown Z Hawrylowicz CM Ligation of TLR9 induced on human IL-10-secreting Tregs by 1alpha,25-dihydroxyvitamin D3 abrogates regulatory function J Clin Invest 2009 119 2 387 398 19139565 10.1172/JCI32354 PMC2631286

[B19] Chambers ES Nanzer AM Richards DF Ryanna K Freeman AT Timms PM Martineau AR Griffiths CJ Corrigan CJ Hawrylowicz CM Serum 25-dihydroxyvitamin D levels correlate with CD4 (+) Foxp3 (+) T-cell numbers in moderate/severe asthma J Allergy Clin Immunol 2012 130 2 542 544 10.1016/j.jaci.2012.04.022 22656048

[B20] Urry ZL Chambers ES Xystrakis E Dimeloe S Richards DF Gabryšová L Christensen J Gupta A Saglani S Bush A O'Garra A Brown Z Hawrylowicz CM The role of 1α, 25-dihydroxyvitamin D3 and cytokines in the promotion of distinct Foxp3+ and IL-10+ CD4+ T cells Eur J Immunol 2012 42 10 2697 2708 10.1002/eji.201242370 22903229 PMC3471131

[B21] Taher YA van Esch BC Hofman GA Henricks PA van Oosterhout AJ 1alpha, 25-dihydroxyvitamin D3 potentiates the beneficial effects of allergen immunotherapy in a mouse model of allergic asthma: role for IL-10 and TGF-beta J Immunol 2008 180 5211 5221 10.4049/jimmunol.180.8.5211 18390702

[B22] Haselden BM Kay AB Larché M Immunoglobulin E-independent major histocompatibility complex-restricted T cell peptide epitope-induced late asthmatic reactions J Exp Med 1999 189 1885 1894 10.1084/jem.189.12.1885 10377184 PMC2192970

[B23] Campbell JD Buckland KF McMillan SJ Kearley J Oldfield WL Stern LJ Grönlund H van Hage M Reynolds CJ Boyton RJ Cobbold SP Kay AB Altmann DM Lloyd CM Larché M Peptide immunotherapy in allergic asthma generates IL-10-dependent immunological tolerance associated with linked epitope suppression J Exp Med 2009 206 7 1535 1547 10.1084/jem.20082901 19528258 PMC2715096

